# Prognostic significance of osteosarcopenia and its effects on immune response in patients with stage II/III gastric cancer

**DOI:** 10.1007/s00262-025-04084-2

**Published:** 2025-05-30

**Authors:** Yuki Hirase, Takaaki Arigami, Daisuke Matsushita, Masataka Shimonosono, Yusuke Tsuruda, Ken Sasaki, Kenji Baba, Yota Kawasaki, Takao Ohtsuka

**Affiliations:** Department of Digestive Surgery, Kagoshima University Graduate School of Medical and Dental Sciences, 8-35-1 Sakuragaoka, Kagoshima, 890-8520 Japan

**Keywords:** Gastric cancer, Osteopenia, Sarcopenia, Osteosarcopenia, Survival, Tumor microenvironment

## Abstract

**Background:**

Osteosarcopenia, characterized by muscle loss and osteoporosis, has emerged as a prognostic marker for various malignancies. However, its impact on the immune response in gastric cancer remains unclear. This study aimed to assess the clinical significance of osteosarcopenia and its relationship with the immune microenvironment in patients with advanced gastric cancer.

**Methods:**

This study included 105 patients with pathological stage II/III gastric cancer who underwent gastrectomy between 2018 and 2022. Preoperative computed tomography was used to measure muscle mass and bone density, with sarcopenia and osteoporosis defined as values below the respective standard thresholds. Sarcopenia and osteoporosis were identified when both conditions were present. We explored the relationships between osteosarcopenia, clinicopathological factors, and prognoses. Additionally, immune marker expression was evaluated via immunohistochemistry.

**Results:**

Among the 105 patients, 37 (35%) were diagnosed with osteosarcopenia. This condition significantly correlated with performance status, body mass index, and disease recurrence (all *p* < 0.05). Overall survival and relapse-free survival were significantly lower in the osteosarcopenia group than those in the non-osteosarcopenia group (all *p* < 0.05). Moreover, the osteosarcopenia group had significantly fewer CD8-positive, programmed cell death protein 1-positive, and programmed death-ligand 1-positive cells than that of the control group (all *p* < 0.05).

**Conclusions:**

Our findings suggest that osteosarcopenia is associated with the tumor microenvironment and might serve as a prognostic indicator in patients with advanced gastric cancer.

## Introduction

Gastric cancer is the fifth most common malignancy worldwide and the fifth leading cause of cancer-related deaths [1]. According to the 2021 Japanese Gastric Cancer Treatment Guidelines (ver. 6), surgery followed by adjuvant chemotherapy is recommended as the standard treatment for patients with stage II/III advanced gastric cancer (AGC) [2]. However, the 5-year survival rate for these patients ranges between 50.2 and 84.2%, indicating a poor prognosis [3]. Therefore, neoadjuvant chemotherapy before gastrectomy can improve the prognosis for patients with stage II/III AGC [4]. However, predicting prognosis before treatment initiation remains clinically challenging. Moreover, no reliable predictors have been closely associated with outcomes in patients with resectable AGC.

Recently, sarcopenia and osteopenia have gained attention as potential prognostic factors [5]. Sarcopenia, characterized by loss of skeletal muscle mass and strength, has been linked to systemic inflammation and impaired immune responses, contributing to poor prognosis in patients with gastric cancer [6]. Similarly, osteopenia, defined as decreased bone mineral density (BMD), has been reported to influence cancer progression through alterations in bone-derived cytokines and the tumor microenvironment (TME), affecting outcomes in several malignancies, including gastric cancer [7, 8]. Both sarcopenia and osteoporosis are significant changes associated with aging, and the combined condition known as “osteosarcopenia” has emerged as a prognostic factor in several cancer types [8, 9]. However, research on osteosarcopenia in patients with AGC is limited, and its clinical impact remains unclear. Furthermore, no studies have assessed osteosarcopenia in the context of the tumor immune microenvironment, leaving its effect on cancer immunity uncertain.

This study investigated sarcopenia, osteopenia, and osteosarcopenia in patients with gastric cancer who underwent gastrectomy with lymphadenectomy. We also evaluated the clinical significance of osteosarcopenia as a novel prognostic marker related to body composition. The immune impact of osteosarcopenia on the TME was also assessed.

## Materials and methods

### Background and purpose

Given the established prognostic relevance of both sarcopenia and osteopenia in patients with gastric cancer, we aimed to elucidate whether the combined condition, osteosarcopenia, may serve as an independent prognostic factor. Furthermore, we performed immunohistochemical analyses to explore the potential association between osteosarcopenia and tumor immunological characteristics.

### Patients

This retrospective study included 134 Japanese patients diagnosed with stage II or III AGC who underwent gastrectomy with lymphadenectomy at Kagoshima University Hospital between January 2018 and December 2022. This period was selected to ensure a sufficient sample size and adequate follow-up time for survival analysis. Among these, 29 patients were excluded owing to the unavailability of preoperative computed tomography (CT) scans at our hospital. Therefore, 105 patients (73 men and 32 women; age range 28–88 years; median age: 66 years) were enrolled. All patients underwent blood tests, esophagogastroduodenoscopy, and CT scans before gastrectomy. Patients were classified and staged according to the tumor-node-metastasis classification of gastric cancer [10].

Power calculation was performed to estimate the required sample size for detecting a significant difference in survival outcomes between patients with and without osteosarcopenia. Assuming a hazard ratio (HR) of 2.0, osteosarcopenia prevalence of 35%, event rate of 30%, two-sided α level of 0.05, and statistical power of 80%, the minimum required sample size was calculated as 84 patients. Therefore, our study cohort of 105 patients was deemed sufficient to detect a statistically significant difference in overall survival.

The “opt-out” method was used to obtain informed consent from the patients. This retrospective study was approved by the Ethics Committee of Kagoshima University (Approval No. 230251).

### Definition of sarcopenia, osteopenia, and osteosarcopenia

Pre-gastrectomy CT images were analyzed using the Volume Analyzer SYNAPSE VINCENT imaging analysis system (Fujifilm Medical, Tokyo, Japan) to assess body composition indices. Sarcopenia was defined as a decrease in the skeletal muscle index (SMI), measured at the L3 vertebral level before gastrectomy [11]. The tissue Hounsfield unit thresholds for skeletal muscle ranged between − 29 and 150 [11]. The SMI (cm^2^/m^2^) was calculated by dividing the skeletal muscle area (cm^2^) by the square of the patient’s height (m^2^) [9]. The cutoff values for SMI were 42 for men and 38 for women [12]. Osteopenia was defined as a condition where the actual BMD was lower than the calculated standard BMD (men: 308.82–2.49 × age [years]; women: 311.84–2.41 × age [years]) [9]. BMD was assessed by examining the average pixel density within an elliptical region of interest at the Th11 vertebral level on preoperative CT images [9]. To improve accuracy, the average pixel density was measured at two locations in different slice planes, from which the average value was calculated. Osteosarcopenia is defined as the coexistence of sarcopenia and osteoporosis. These definitions were adopted from previously published and widely cited studies in which body composition and cancer prognosis in patients with gastrointestinal malignancies were investigated [9, 11, 12]. Alternative criteria, such as those established by the Asian Working Group for Sarcopenia (AWGS), are available and more specific to Asian populations; however, this retrospective study did not include data on muscle strength or physical performance, which are required to apply AWGS criteria [13]. Therefore, CT-based classification using SMI was employed. Similarly, owing to the retrospective nature of this study, BMD measurements using dual-energy X-ray absorptiometry (DXA), the current gold standard, were unavailable, and BMD was estimated using Hounsfield units from CT images.

### Blood markers for nutrition and systemic inflammatory response

In this study, neutrophil-to-lymphocyte ratio (NLR), prognostic nutritional index (PNI), C-reactive protein (CRP)-to-albumin ratio (CAR), and albumin levels were used as blood markers for nutritional and systemic inflammatory responses. Blood samples were collected before gastrectomy. Neutrophils, lymphocytes, and platelets were counted using an XN-20 automated hematology analyzer (Sysmex Corporation, Kobe, Japan). CRP and albumin levels were measured using a JCA-ZS050 automated analyzer (JEOL Ltd., Tokyo, Japan).

NLR was calculated by dividing the number of neutrophils by the number of lymphocytes [14]. PNI was determined as serum albumin (g/L) × 10 + 0.005 × total lymphocyte count (per mm^3^) [14]. CAR was calculated by dividing CRP by the albumin level [14]. The cutoff values for NLR, PNI, CAR, and albumin levels in the univariate and multivariate analyses were set using the median values.

### Immunohistochemistry (IHC)

Tumor samples were obtained from specimens resected during surgery. They were fixed in 10% formaldehyde in phosphate-buffered saline, embedded in paraffin, and cut into 4-mm-thick slices. After deparaffinization, the sections were pretreated by immersion in citrate buffer and heated in a microwave at 121 °C for 10 min. The sections were washed twice with distilled water for 5 min each and then immersed in a 0.3% hydrogen peroxide/methanol solution at room temperature for 10 min to block endogenous peroxidase activity. For staining with antibodies against CD4, CD8, forkhead box P3 (FOXP3), programmed cell death protein 1 (PD-1), and programmed death-ligand 1 (PD-L1), the sections were blocked with 2.5% normal horse serum for 30 min. The blocked sections were incubated with primary antibodies diluted in Antibody-Diluent at 4 °C for 2 h, followed by staining using the ImmPRESS-HRP Reagent Kit (Vector Laboratories, Newark, CA, USA). The primary antibodies and their dilutions were as follows: anti-CD4 (ab133616; Abcam, Cambridge, UK; 1:500), anti-CD8 (M7103; Agilent Dako, Glostrup, Denmark; 1:100), anti-FOXP3 (ab20034; Abcam; 1:500), anti-PD-1 (ab137132; Abcam; 1:500), and anti-PD-L1 (13684S; Cell Signaling Technology, Danvers, MA, USA; 1:200). The sections were washed twice in Tris-buffered saline with Tween 20 for 5 min each and then incubated with diaminobenzidine tetrahydrochloride to visualize the immune complexes. Finally, the sections were lightly rinsed with water, counterstained with hematoxylin, and mounted.

IHC observations were performed using an Olympus BX53 optical microscope (Olympus, Tokyo, Japan) equipped with the cellSens standard software (Olympus) and digitized. Images were captured at five arbitrarily selected hotspots at 400 × magnification. Image files with a resolution of 1920 × 1080 pixels were saved in the JPEG format.

IHC image analysis was performed using ImageJ software, version 1.53t (NIH, Bethesda, MD, USA). The IHC Profiler plugin utilized color deconvolution to measure the antigen expression intensity. The resulting images included color 1, representing hematoxylin staining, and color 2, representing 3,3′-diaminobenzidine (DAB) staining. Color 3 was not necessary for this analysis. Segmentation was based on color 1, and the number of cells expressing CD4, CD8, FOXP3, PD-1, and PD-L1 was evaluated based on color 2. The intensity of the DAB color was calculated, with the analyzed parameter being the mean gray value, which ranged between 0 and 255; 0 represented the darkest color, while 255 represented the lightest. The test results were categorized based on the classification provided by IHC Profiler. Mean gray values were classified as positive (strong: 0–60, moderate: 61–120, weak: 121–180) and negative for values over 180 [15]. The number of positive cells in the five hotspots was counted, and the average value was calculated. PD-L1 expression was analyzed by distinguishing between tumor cells and peritumoral immune cells. Tumor cells were identified based on nuclear morphology and cell boundaries, whereas immune cells were distinguished as small mononuclear cells with high nuclear density. Only PD-L1-positive tumor cells were counted, and instead of using clinically established scoring systems such as the Tumor Proportion Score or Combined Positive Score [16], the evaluation was based solely on the number of positive cells.

To ensure consistency and reproducibility, the threshold settings used in the analysis were standardized across all samples. The IHC Profiler plugin was used with default threshold ranges corresponding to the strong, moderate, and weak positivity categories. Additionally, interobserver variability was assessed by two independent observers (Y.H. and T.A.) analyzing a random subset of 10 images. The interclass correlation coefficient was calculated as > 0.85, indicating excellent agreement.

### Statistical analyses

The relationships between the presence of osteosarcopenia and clinicopathological factors, as well as between osteosarcopenia and tumor immunohistochemistry, were evaluated using the chi-square test, Fisher’s exact test, and Wilcoxon rank-sum test. Overall survival (OS) was defined as the period from surgery to death or last follow-up. Relapse-free survival (RFS) was defined as the period from surgery to the date of confirmed recurrence. Kaplan–Meier survival curves were generated, and prognostic differences were determined using the log-rank test. Prognostic factors were determined using univariate and multivariate analyses with Cox proportional hazards regression modeling. All data were analyzed using JMP software (SAS Institute Inc., Cary, NC, USA), and significance was determined only for factors with *p* < 0.05, which is a widely accepted threshold in clinical research for determining whether an observed effect is unlikely to have occurred by chance. *p* < 0.01 indicates a stronger level of statistical significance; however, using *p* < 0.05 as the cutoff allows for a balanced interpretation of the results, particularly in exploratory or observational studies with relatively small sample sizes. We acknowledge that *p*-values below 0.01 suggest more robust associations and have indicated such findings accordingly where applicable.

## Results

### Clinicopathological features of patients

The clinicopathological characteristics of the 105 patients are summarized in Table 1. The performance status (PS) of the patients was as follows: 80 patients (76%) had PS 0, 19 (18%) had PS 1, three (3%) had PS 2, and three (3%) had PS 3. The median body mass index (BMI) was 21.4 kg/m^2^ (range 15.4–30.6 kg/m^2^). A total of 44 patients (42%) received preoperative chemotherapy, and 104 patients (99%) received postoperative adjuvant chemotherapy. The types of surgery were as follows: total gastrectomy in 53 patients (50%), proximal gastrectomy in 11 (10%), distal gastrectomy in 37 (35%), partial gastrectomy in 2 (2%), esophagectomy in 1 (1%), and pancreatoduodenectomy in 1 (1%). The median clinical tumor size was 60.5 mm (range 16–230 mm).

**Table 1:** Clinicopathological factors of patients (*n* = 105)

Factor	*n*
Sex, male/female	73/32
Median age (range), years	66 (28–88)
Performance status, 0/1/2/3	80/19/3/3
Body mass index (range), kg/m^2^	21.4 (15.4–30.6)
Preoperative chemotherapy, presence/absence	44/61
Adjuvant chemotherapy, presence/absence	104/1
Surgical method of surgery	
total gastrectomy/proximal gastrectomy/distal gastrectomy/partial gastrectomy/esophagectomy/pancreaticoduodenectomy	53/11/37/2/1/1
Median clinical tumor size (range), mm	60.5 (16–230)
Depth of tumor invasion, pT1/pT2/pT3/pT4	2/5/48/50
Lymph node metastasis, pN0/pN1/pN2/pN3	27/20/30/28
Pathological stage, II/III	43/62
Median CEA (range)	2.7 (0.4–426)
Median CA19-9 (range)	11 (0.9–3314)
Median NLR (range)	2.32 (0.30–12.14)
Median PNI (range)	46.81 (32.14–893.46)
Median CAR (range)	0.03 (0.01–3.53)
Median albumin (range), g/dL	3.9 (2.5–5.3)

CEA, carcinoembryonic antigen; CA19-9, carbohydrate antigen 19-9; NLR, neutrophil–lymphocyte ratio; PNI, prognostic nutritional index; CAR, C-reactive protein–albumin ratio

Regarding the depth of tumor invasion, two patients (2%) had pathological T1 tumors, five (5%) had T2 tumors, 48 (46%) had T3 tumors, and 50 (48%) had T4 tumors. Additionally, 27 patients (26%) had pathological N0 disease, 20 (20%) had N1 disease, 30 (29%) had N2 disease, and 28 (27%) had N3 disease. Overall, 43 patients (41%) had stage II disease, and 62 (59%) had stage III disease.

The median preoperative values for carcinoembryonic antigen, carbohydrate antigen (CA) 19-9, NLR, PNI, CAR, and albumin were 2.7 ng/mL (range 0.4–426 ng/mL), 11 U/mL (range 0.9–3314 U/mL), 2.32 (range 0.30–12.14), 46.81 (range 32.14–893.46), 0.03 (range 0.01–3.53), and 3.9 g/dL (range 2.5–5.3 g/dL), respectively.

### Assessment of sarcopenia, osteopenia, and osteosarcopenia

**Fig. 1:**
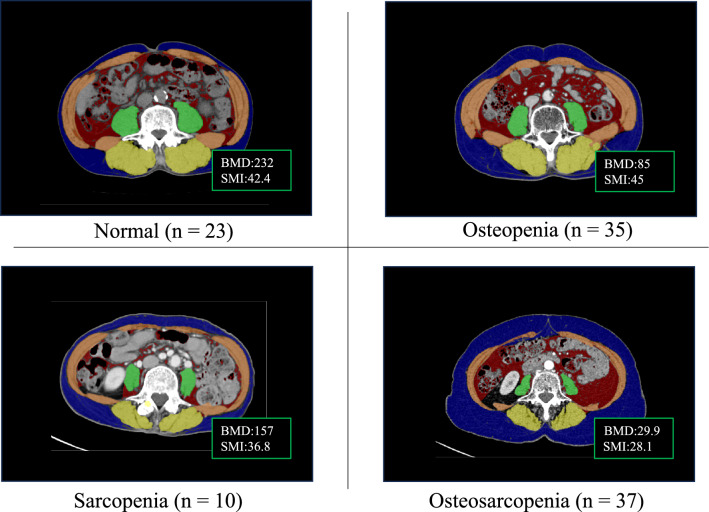
Representative computed tomography images of patients with normal, sarcopenia alone, osteopenia alone, and osteosarcopenia

Figure 1 shows representative CT images of patients with normal weight, sarcopenia, osteopenia, and osteosarcopenia. Twenty-three (22%), 10 (10%), 35 (33%), and 37 (35%) patients had normal weight, sarcopenia alone, osteopenia alone, and osteosarcopenia, respectively.

### Relationship between osteosarcopenia and clinicopathological factors

The presence of osteosarcopenia was significantly associated with PS, BMI, and recurrence (*p* = 0.01, *p* = 0.01, and *p* < 0.01, respectively; Table 2). No significant differences were observed between osteosarcopenia and other characteristics, such as sex, age, preoperative chemotherapy, surgical procedure, tumor size, tumor invasion depth, lymph node metastasis, pathological stage, carcinoembryonic antigen, CA19-9, NLR, PNI, CAR, and albumin (all *p* > 0.05; Table 2).

**Table 2:** Relationship between the presence or absence of osteosarcopenia and clinicopathological factors in all patients (*n* = 105)

Factor	Osteosarcopenia, *n* (%)		
	Presence (*n* = 37)	Absence (*n* = 68)	*p*-value
Sex, male/female	23/14	50/18	0.22
Median age, years	69	65	0.13
Performance status, 0/1–3	23/14	57/11	0.01
Median Body mass index, kg/m^2^	20.5	21.85	0.01
Preoperative chemotherapy, presence	24	37	0.29
Surgical method of surgery, total gastrectomy, presence	17	36	0.54
Median clinical tumor size (range), mm	65	60	0.15
Depth of tumor invasion, pT1/pT2–4	1/36	1/67	0.18
Lymph node metastasis, pN0/pN1–3	6/31	21/47	0.10
Pathological stage, II/III	12/25	31/37	0.21
Median CEA	2.7	2.7	0.06
Median CA19-9	11.6	9.9	0.89
Median NLR	2.95	2.18	0.25
Median PNI	45.3	47.5	0.40
Median CAR	0.03	0.03	0.31
Median albumin, g/dL	3.8	3.9	0.45
Recurrence, presence	30	32	< 0.01

CEA, carcinoembryonic antigen; CA19-9, carbohydrate antigen 19-9; NLR, neutrophil–lymphocyte ratio; PNI, prognostic nutritional index; CAR; C-reactive protein albumin ratio

### Prognostic analysis determined by osteosarcopenia

**Fig. 2:**
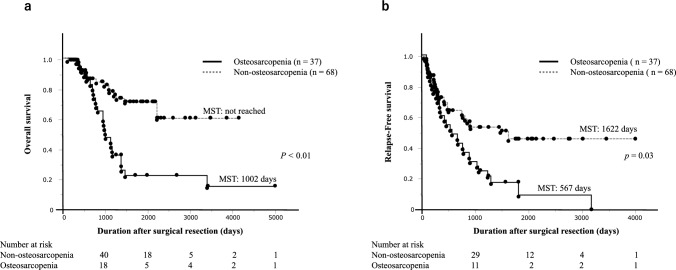
Kaplan–Meier survival curves after surgical resection in patients with osteosarcopenia and non-osteosarcopenia. **a** Overall survival; **b** relapse-free survival

The median OS of the 105 cases was 2217 days. The median OS was significantly shorter in the osteosarcopenia group than in those of the non-osteosarcopenia group (1002 days vs. not reached; *p* < 0.01) (Fig. 2a). Univariate analysis showed that PS (0 vs. 1–3), lymph node metastasis (pN0–2 vs. pN3), NLR, and osteosarcopenia were significantly associated with OS (*p* < 0.01, *p* < 0.01, *p* = 0.04, and *p* < 0.01, respectively; Table 3). In the multivariate analysis, PS (0 vs. 1–3), lymph node metastasis (pN0–2 vs. pN3), NLR, and osteosarcopenia were identified as independent prognostic factors for OS (*p* = 0.02, *p* < 0.01, *p* = 0.03, and *p* = 0.03, respectively; Table 3).

**Table 3:** Univariate and multivariate analysis of overall survival in patients with gastric cancer undergoing surgical resection (*n* = 105)

Independent factor	Univariate analysis			Multivariate analysis		
	Hazard ratio	95% CI	*p-*value	Hazard ratio	95% CI	*p-*value
Gender, male	0.74	0.39–1.41	0.37			
Age, ≧ 66 years	1.45	0.79–2.64	0.22			
Performance status, 1–3	5.10	2.67–9.64	< 0.01	2.41	1.13–5.15	0.02
Body mass index, < 21.4 kg/m^2^	1.52	0.83–2.80	0.17			
Preoperative chemotherapy, absence	1.54	0.85–2.81	0.16			
Surgical method of surgery, total gastrectomy, presence	0.84	0.46–1.52	0.56			
Depth of tumor invasion, pT2–4	1.02	0.14–7.49	0.97			
Lymph node metastasis, pN3	3.16	1.69–5.95	< 0.01	3.68	1.81–7.48	< 0.01
Pathological stage, III	1.79	0.93–3.43	0.07			
CEA, > 5.0	0.96	0.46–2.02	0.93			
CA19-9, > 37.0	1.87	0.98–3.59	0.06			
NLR, ≧ 2.32	1.86	1.00–3.43	0.04	2.11	1.08–4.13	0.03
PNI, < 46.75	1.51	0.83–2.77	0.17			
CAR, ≧ 0.03	1.06	0.56–2.01	0.83			
Albumin, < 3.9 g/dL	1.63	0.89–2.96	0.10			
Osteosarcopenia, presence	3.33	1.80–6.14	< 0.01	2.16	1.05–4.43	0.03

CEA, carcinoembryonic antigen; CA19-9, carbohydrate antigen 19-9; NLR, neutrophil–lymphocyte ratio; PNI, prognostic nutritional index; CAR, C-reactive protein–albumin ratio

The median RFS of 105 patients was 809 days. The median RFS was significantly shorter in the osteosarcopenia group compared to those in the non-osteosarcopenia group (567 vs. 1622 days; *p* = 0.03; Fig. 2b). In the univariate analysis, PS (0 vs. 1–3), lymph node metastasis (pN0–2 vs. pN3), NLR, and osteosarcopenia were significant prognostic factors for RFS (*p* < 0.01, *p* < 0.01, *p* = 0.02, and *p* < 0.01, respectively; Table 4). In multivariate analysis, PS (0 vs. 1–3) and lymph node metastasis (pN0–2 vs. pN3) were identified as independent prognostic factors for RFS (*p* = 0.02 and *p* < 0.01, respectively; Table 4).

**Table 4:** Univariate and multivariate analysis of relapse-free survival in patients with gastric cancer undergoing surgical resection (*n* = 105)

Independent factor	Univariate analysis			Multivariate analysis		
	Hazard ratio	95% CI	*p-*value	Hazard ratio	95% CI	*p-*value
Gender, male	1.01	0.57–1.77	0.95			
Age, ≧ 66 years	1.06	0.64–1.76	0.80			
Performance status, 1–3	3.67	2.14–6.29	< 0.01	2.16	1.12–4.17	0.02
Body mass index, < 21.4 kg/m^2^	1.50	0.89–2.51	0.12			
Preoperative chemotherapy, absence	1.21	0.64–2.29	0.55			
Surgical method of surgery, total gastrectomy, presence	0.85	0.51–1.41	0.55			
Depth of tumor invasion, pT2–4	1.76	0.24–12.77	0.57			
Lymph node metastasis, pN3	2.43	1.43–4.14	< 0.01	2.33	1.30–4.17	< 0.01
Pathological stage, III	1.45	0.84–2.48	0.17			
CEA, > 5.0	1.57	0.88–2.79	0.13			
CA19-9, > 37.0	1.40	0.78–2.50	0.24			
NLR, ≧ 2.32	1.83	1.08–3.09	0.02	1.67	0.96–2.87	0.06
PNI, < 46.75	1.36	0.81–2.27	0.23			
CAR, ≧ 0.03	1.20	0.71–2.03	0.49			
Albumin, < 3.9 g/dL	1.65	0.99–2.75	0.05			
Osteosarcopenia, presence	2.14	1.29–3.55	< 0.01	1.51	0.84–2.74	0.17

CEA, carcinoembryonic antigen; CA19-9, carbohydrate antigen 19-9; NLR, neutrophil–lymphocyte ratio; PNI, prognostic nutritional index; CAR, C-reactive protein–albumin ratio

### Subgroup prognostic analysis determined by sarcopenia, osteopenia, and osteosarcopenia

To further evaluate the significance of preoperative body composition in patients with AGC, the patients were categorized into three groups: normal (*n* = 23), sarcopenia or osteopenia alone (n = 45), and osteosarcopenia (*n* = 37).

**Fig. 3:**
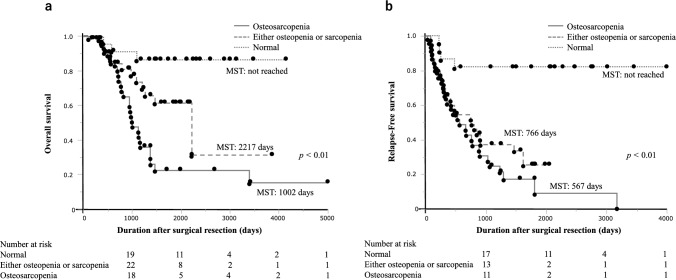
Kaplan–Meier survival curves after surgical resection in patients with normal, either sarcopenia or osteopenia alone, and osteosarcopenia. **a** Overall survival; **b** relapse-free survival

A significant difference in the median OS was observed among the three groups (median OS: not reached, 2217 days, and 1002 days; *p* < 0.01; Fig. 3a). A significant difference in the median RFS was observed among the three groups (median RFS: not reached, 766 days, and 567 days; *p* < 0.01; Fig. 3b).

### Relationship between osteosarcopenia and immune microenvironments

**Fig. 4:**
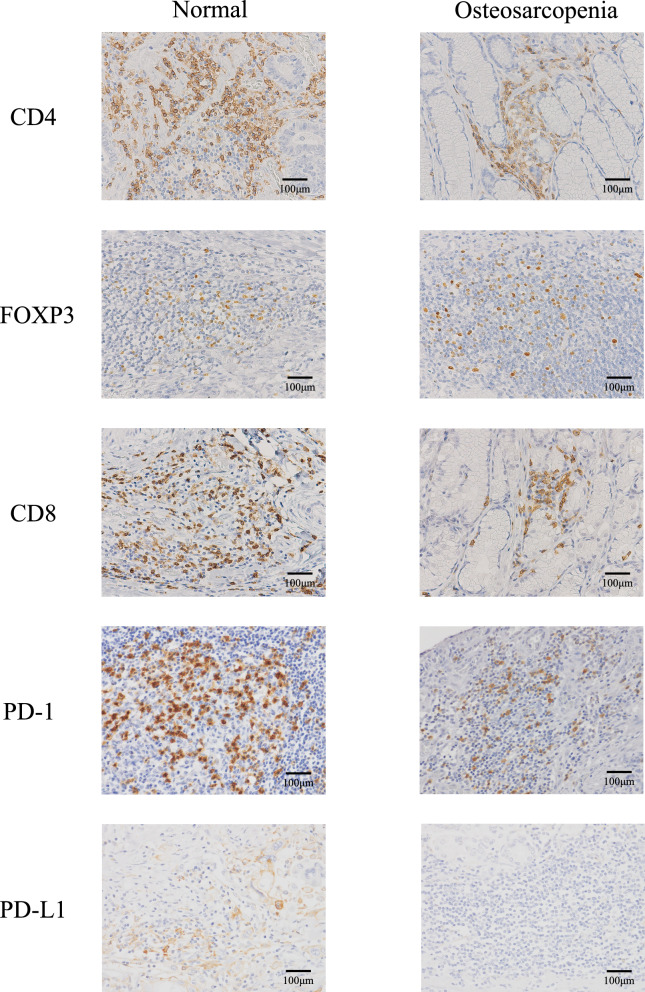
Positive immunostaining for representative CD4, FOXP3, CD8, PD-1, and PD-L1 in patients with normal status and osteosarcopenia

**Fig. 5:**
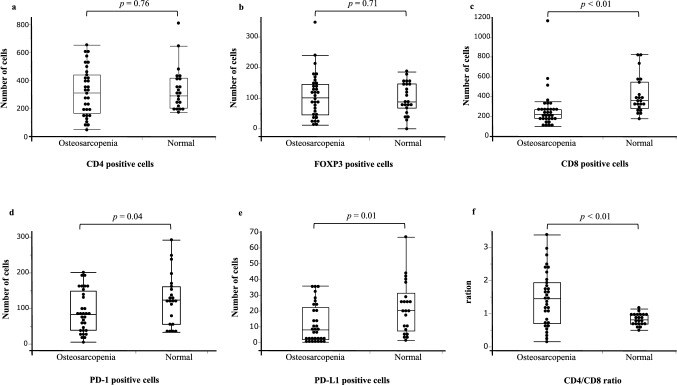
Comparison of immunostaining between normal patients (n = 23) and those with osteosarcopenia (n = 37). The CD4-positive cell count (**a**) and FOXP3-positive cell count (**b**) were not significantly different between the two groups. However, the numbers of CD8-positive cells (**c**), PD-1-positive cells (**d**), and PD-L1-positive cells (**e**) were significantly lower in patients with osteosarcopenia. The CD4/CD8 ratio (**f**) was significantly higher in patients with osteosarcopenia

Immunostaining was performed on resected specimens from 23 healthy participants, excluding patients with osteopenia alone and sarcopenia alone, and 37 patients with osteosarcopenia by comparing the numbers of CD4-, CD8-, FOXP3-, PD-1, and PD-L1-positive cells between the groups. Figure 4 shows representative images of patients with normal and osteosarcopenia. There were no significant differences in the number of CD4-positive cells or FOXP3-positive cells between patients with normal and those with osteosarcopenia (*p* = 0.76 and *p* = 0.71, respectively; Fig. 5a, b). However, the numbers of CD8-positive, PD-1-positive, and PD-L1-positive cells were significantly lower in patients with osteosarcopenia than in those with normal patients (*p* < 0.01, *p* = 0.04, and *p* = 0.01, respectively; Fig. 5c–e). Additionally, the CD4/CD8 ratio was significantly higher in patients with osteosarcopenia than in healthy participants (*p* < 0.01; Fig. 5f).

## Discussion

This study evaluated the clinical impact of osteosarcopenia in patients with AGC who underwent surgical resection, using body composition and immunohistochemistry analyses. The following results were obtained: (1) A significant relationship was observed between osteosarcopenia and PS, BMI, and postoperative recurrence. (2) Multivariate analysis identified osteosarcopenia, PS, lymph node metastasis, and NLR as independent prognostic factors for OS. (3) Subgroup analysis showed that median OS and RFS were significantly lower in patients with osteosarcopenia than in those with either sarcopenia or osteopenia alone. (4) Immunohistochemistry revealed significantly lower numbers of CD8-positive, PD-1-positive, and PD-L1-positive cells in the osteosarcopenia group than in those of the healthy group. To the best of our knowledge, this is the first study to suggest that osteosarcopenia is related to TME and demonstrates its potential to predict prognosis in patients with stage II/III AGC undergoing gastrectomy.

Patients with advanced cancer often experience malabsorption and malnutrition, which lower PS and BMI, contributing to osteopenia and sarcopenia [17]. Cancer metastasis often occurs in the bone marrow microenvironment with reduced bone density, and sarcopenia is closely related to tumor formation and cancer cell proliferation [18, 19]. In this study, the osteosarcopenia group exhibited a higher recurrence rate than those of the non-osteosarcopenia group, suggesting an association between osteosarcopenia and malignant aggressiveness.

Sarcopenia is associated with cancer-induced obstruction, malabsorption, inflammation due to cytokine release, and reduced immune activity [20]. Osteopenia is also reported to be induced by cancer-related inflammatory cytokines, which lead to osteoclast formation [21]. These findings associate various inflammatory markers with the prognosis of patients with gastric cancer [22]. In this study, NLR, PNI, CAR, and serum Alb levels were investigated regarding gastric cancer prognosis, and a significant association with NLR was found—consistent with previous reports [22]. NLR showed a significant difference for OS in both univariate and multivariate analyses.

Researchers have explored the clinical impact of osteosarcopenia as a prognostic factor in patients with malignant tumors [23]. Matsumoto et al. investigated the prognosis of 138 patients with extrahepatic bile duct cancer after resection and reported 5-year OS rates of 20.5% in the osteosarcopenia group compared to 67.8% in that of non-osteosarcopenia group (*p* < 0.0001) [5]. In this study, OS and RFS were significantly shorter in the osteosarcopenia than in those of non-osteosarcopenia group. Multivariate analysis identified osteosarcopenia as an independent prognostic factor for OS related to body composition. Furthermore, osteosarcopenia remained a significant independent prognostic factor for overall survival in the multivariate Cox proportional hazards model that included nutritional and inflammatory markers such as BMI and serum albumin (coefficient = 0.58, HR = 3.21, *p* < 0.01). These findings indicate that osteosarcopenia is strongly associated with poor prognosis, even after adjusting for nutritional status. Notably, osteopenia and sarcopenia have been linked to the prognosis of patients with gastric cancer [24, 25]. Our subgroup analysis demonstrated that osteosarcopenia, a combination of both conditions, was a stronger prognostic factor than either osteopenia or sarcopenia alone. Therefore, osteosarcopenia may be a promising surrogate marker for predicting the prognosis of patients with advanced stage II/III AGC.

IHC was performed to investigate the relationship between osteosarcopenia and TME. Notably, patients with osteosarcopenia had significantly fewer CD8-, PD-1-, and PD-L1-positive cells than healthy controls. TME is crucial for the response to immune checkpoint inhibitors (ICIs) [26]. CD8-positive cells are key effector cells in the anticancer immune process, and their quantity and cytotoxic potential significantly influence immunotherapy outcomes [27, 28]. When CD8-positive cell proliferation decreases and cytotoxic molecules diminish, CD8-positive cell exhaustion occurs, leading to reduced expression of PD-1 and PD-L1 and increased resistance to ICIs [29, 30]. This exhaustion is often associated with immune-suppressive cytokines such as transforming growth factor beta and interleukin-10, which further impair the function of CD8-positive cells. Tumors in this state are termed “cold tumors,” and studies have been conducted to convert them into “hot tumors,” which are more responsive to ICI therapy [31, 32]. Sarcopenia and osteopenia have been linked to CD8-positive cell exhaustion [33, 34]. Osteosarcopenia, which is the combination of both conditions, may further worsen the reduction of CD8-positive cells and is likely associated with cold tumors. Low PD-L1 expression is generally associated with cold tumors [35]; however, high PD-L1 expression has also been linked to immune evasion in certain contexts [36], making it important to interpret PD-L1 levels carefully when evaluating immune escape mechanisms. These findings indicate that osteosarcopenia may influence the tumor immune microenvironment. Future studies incorporating functional markers such as Granzyme B and multiplex IHC could strengthen these conclusions by providing further insights into the cytotoxic potential of immune cells and spatial immune cell distributions within the TME. Therefore, the presence or absence of osteosarcopenia could help predict the clinical efficacy of ICI therapy in patients with stage IV or recurrent gastric cancer.

Pharmacotherapy, exercise therapy, and nutritional programs may help treat sarcopenia and osteopenia in patients with malignancies [37]. Vitamin D deficiency is a significant risk factor for low BMD. Therefore, preoperative pharmacological support, including calcium intake and vitamin D supplementation, may help prevent osteopenia [38]. Calcium, vitamin D, and bisphosphonates interventions have been shown to increase BMD in patients with gastric cancer after gastrectomy, thereby reducing fracture risk [39]. Exercise and nutritional programs may help prevent sarcopenia by increasing protein synthesis [40]. However, to our knowledge, no prospective studies have demonstrated that perioperative interventions involving pharmacotherapy, nutritional support, and rehabilitation improve postoperative outcomes in patients with gastric cancer. Further research is needed to assess whether interventions targeting osteosarcopenia can positively affect prognosis in clinical management.

This study had some limitations. First, it was a single-center retrospective analysis with a relatively small sample size (n = 105), which may limit the statistical power and generalizability of the findings. Second, the study population consisted exclusively of Japanese patients, with sex imbalance, representing a single ethnic group, which may limit the applicability of the results to more diverse populations. Third, owing to the limited number of patients, including the normal group (n = 23), the findings may not be generalizable to all individuals with gastric cancer. Fourth, BMD was assessed using CT-based methods instead of the gold-standard DXA, which may have limited the diagnostic accuracy. Finally, chemotherapy regimens and dose modifications were determined based on clinical trial protocols, individual patient conditions, or physician discretion, potentially introducing treatment-related biases that could have influenced the study outcomes.

Overall, this study suggests that osteosarcopenia may be associated with the TME and could serve as a potential factor for predicting the prognosis of patients with stage II/III AGC, warranting further investigation.

## Data Availability

No datasets were generated or analysed during the current study.
